# Supplementary material for: Serum Insufficiency Induces RANKL-Independent Osteoclast Formation during Developing Ischemic ONFH
Source: Biomedicines. 2021 Jun 17;9(6):685. doi: 10.3390/biomedicines9060685 (PMC8234979; doi:10.3390/biomedicines9060685)
Supplement: Supplementary file 1 [file biomedicines-09-00685-s001.zip › biomedicines-1237907-supplementary.pdf]

biomedicines

MDPI

Article

Serum Insufficiency Induces RANKL-Independent Osteoclast Formation during Developing Ischemic ONFH

Yi-min Hsiao [1,2], Chih-Chien Hu [1,2], Mei-Feng Chen [2], Chih-Hsiang Chang [1,2,3,4], Yu-Tien Chiu [2] and Yuhan Chang [1,2,*]

Supplementary Materials

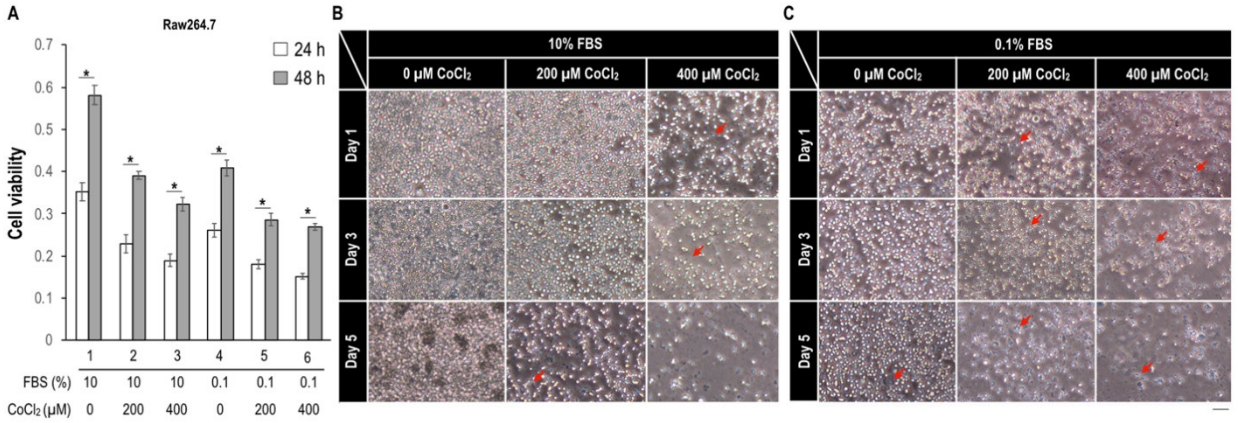

Figure S1. Proliferation of monocytes and preosteoclasts subjected to serum deprivation, hypoxia, or both. a Proliferation of RAW 264.7 cells. Groups 1–3 are serum-rich (10% FBS) groups; Groups 4–6 are serum-deprived (0.1% FBS) group. Groups 1 and 4 are vehicle controls. Groups 2 and 5 are 200 μM $CoCl_2$-treated group. Groups 3 and 6 are 400 μM $CoCl_2$-treated group. The white bar indicates 24-h treatment, and the gray bar indicates 48-h treatment. Each bar corresponds to a single group represented as mean ± SD (n = 6). Serum-deprived, hypoxic, and ischemic condition repressed cell proliferation. Asterisks indicate $p < 0.05$. **b** Morphology and cell density of monocytes after treating 10% FBS combined without and with $CoCl_2$ treatment. c) Morphology and cell density of monocytes after treating 0.1% FBS combined without and with $CoCl_2$ treatment. Arrows indicate preosteoclast-like cells. Cell density was reduced after serum deprivation, $CoCl_2$ treatment, and both. Abbreviations: FBS, fetal bovine serum; h, hour. Bar scale: 100 μm.

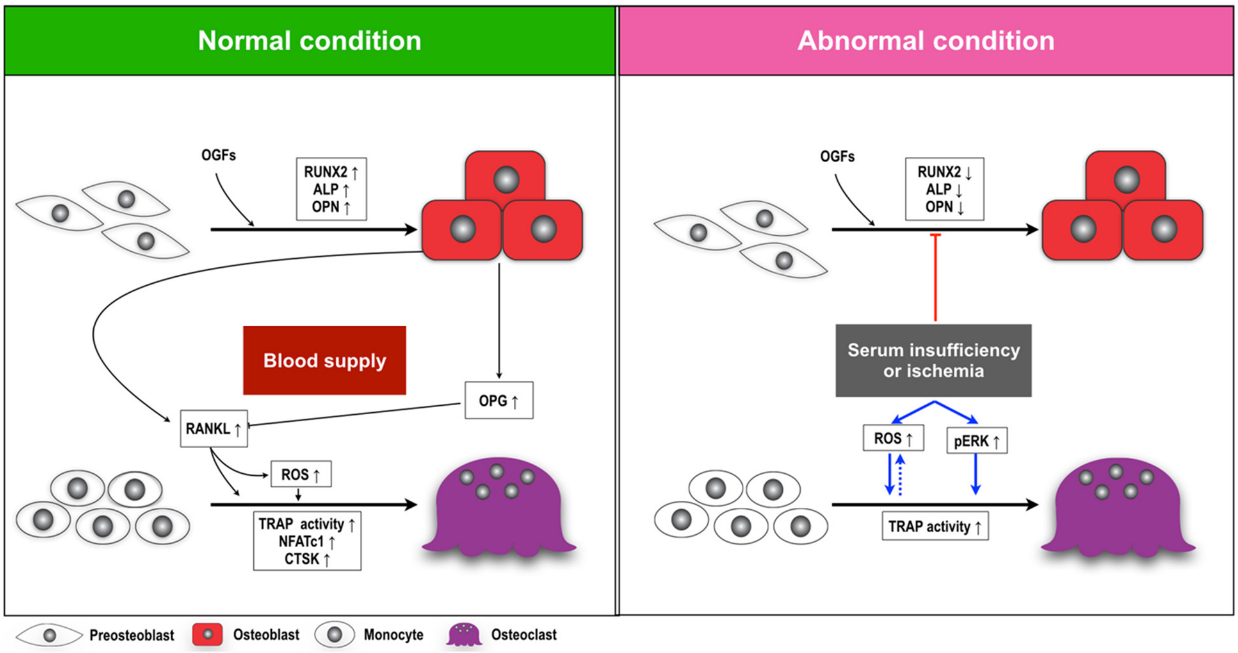

Figure S2. Graphic summary of the hypothesized mechanisms of avascular osteonecrosis. Black lines indicate information have been reviewed[1], and colored lines indicate the findings of this study, and boxes with dashed lines indicate that the factors is unverified. In normal development (serum and oxygen sufficiency), osteoblast and osteoclast communication reveals a balance in their activity. Under conditions of serum deprivation or hypoxia, osteoclast activity is increased and osteoblast activity is inhibited. Notably, serum insufficiency, as well as hypoxia, can increase the formation of TRAP-positive preosteoclasts and trigger cell fusion for TRAP-positive osteoclast formation. The results also indicate that an ischemic condition inhibited the expression of osteoblast proteins (ALP, RUNX2, and OPN) and increased the abundance of osteoclast TRAP protein, rather than CTSK and NFATc1. Although the hierarchical and molecular factors – RUNX2, ROS, and pERK1/2 or mechanisms of serum insufficiency/ischemia–induced higher TRAP activity and osteoclast formation remain unclear, these results suggest that may cause the acceleration of osteoclast formation in *in vitro* and in the patients with ONFH (pERK and TRAP were highly expressed under ischemia). Abbreviations: CTSK, cathepsin K; OGFs, osteogenic growth factors; OPG, osteoprotegerin; OPN, osteopontin; ROS, reactive oxygen species; pERK1/2, phosphorylated extracellular-regulated kinase 1/2; RUNX2, runt-related transcription factor 2; TRAP, tartrate-resistant acid phosphatase.

Table S1. The list of primary and secondary antibodies and working dilutions in this study.

Primary Ab	Dilution	Catalog number	Secondary Ab	Dilution	Catalog number
ALP	1:250	sc-80678	Anti-mouse IgG, HRP-linked	1:1000	CS #7076
β-actin	1:10000	sc-47778		1:10000	
CTSK	1:1000	sc-48353		1:1000	
NFATc1	1:1000	sc-7294		1:1000	
OPN	1:1000	sc-21742		1:1000	
RUNX2	1:4000	CS #12556	Anti-rabbit IgG, HRP-linked	1:2000	CS #7074
TRAP	1:4000	ab191406		1:2000	
ERK1/2	1:2000	CS #9102		1:4000	
pERK1/2	1:2000	CS #4370		1:4000	

Abbreviations: Ab, antibody; ab, Abcam; CS, Cell Signaling; sc, Santa Cruz.

Reference

1. Chen, X.; Wang, Z.; Duan, N.; Zhu, G.; Schwarz, E.M.; Xie, C. Osteoblast-osteoclast interactions. *Connect Tissue Res.* **2018**, *59*, 99-107, doi:10.1080/03008207.2017.1290085.
